# Expression of huntingtin-associated protein 1 in adult mouse dorsal root ganglia and its neurochemical characterization in reference to sensory neuron subpopulations

**DOI:** 10.1016/j.ibror.2020.10.001

**Published:** 2020-10-06

**Authors:** Md Nabiul Islam, Naoki Maeda, Emi Miyasato, Mir Rubayet Jahan, Abu Md Mamun Tarif, Taiga Ishino, Kanako Nozaki, Koh-hei Masumoto, Akie Yanai, Koh Shinoda

**Affiliations:** a Division of Neuroanatomy, Department of Neuroscience, Yamaguchi University Graduate School of Medicine, 1-1-1 Minami-Kogushi, Ube, 755-8505, Japan; b Department of Anatomy and Histology, Faculty of Veterinary Science, Bangladesh Agricultural University, Mymensingh, 2202, Bangladesh; c Department of Basic Laboratory Sciences, Faculty of Medicine and Health Sciences, Yamaguchi University Graduate School of Medicine, 1-1-1 Minami-Kogushi, Ube, 755-8505, Japan

**Keywords:** CB, calbindin, CGRP, calcitonin gene-related peptide, CR, calretinin, DAB, diaminobenzidine, DRG, dorsal root ganglia, HAP1, Huntingtin-associated protein 1, htt, huntingtin, Iba1, ionized calcium-binding adapter molecule 1, LTMRs, low-threshold mechanoreceptors, MRGPR, Mas-related G-protein-coupled receptor, NDS, normal donkey serum, NOS, nitric oxide synthetase, NeuN, neuronal nuclei, PB, phosphate buffer, polyQ, polyglutamine, PV, parvalbumin, SBMA, spinal and bulbar muscular atrophy, STB, stigmoid body, SP, substance P, TBST, Tris-buffered saline with 0.1 % Tween, TH, tyrosine hydroxylase, TRPV1, transient receptor potential vanilloid 1, VGLUT, vesicular glutamate transporter, Huntingtin-associated protein 1, Peripheral nervous system, Sensory neurons, Neuroprotection, Neurodegeneration, Immunohistochemistry

## Abstract

Huntingtin-associated protein 1 (HAP1) is a polyglutamine (polyQ) length-dependent interactor with causal agents in several neurodegenerative diseases and has been regarded as a protective factor against neurodegeneration. In normal rodent brain and spinal cord, HAP1 is abundantly expressed in the areas that are spared from neurodegeneration while those areas with little HAP1 are frequent targets of neurodegeneration. We have recently showed that HAP1 is highly expressed in the spinal dorsal horn and may participate in modification/protection of certain sensory functions. Neurons in the dorsal root ganglia (DRG) transmits sensory stimuli from periphery to spinal cord/brain stem. Nevertheless, to date HAP1 expression in DRG remains unreported. In this study, the expression of HAP1 in cervical, thoracic, lumbar and sacral DRG in adult male mice and its relationships with different chemical markers for sensory neurons were examined using Western blot and immunohistochemistry. HAP1-immunoreactivity was detected in the cytoplasm of DRG neurons, and the percentage of HAP1-immunoreactive (ir) DRG neurons was ranged between 28–31 %. HAP1-immunoreactivity was comparatively more in the small cells (47–58 %) and medium cells (40–44 %) than that in the large cells (9–11 %). Double-immunostaining for HAP1 and markers for nociceptive or mechanoreceptive neurons showed that about 70–80 % of CGRP-, SP-, CB-, NOS-, TRPV1-, CR- and PV-ir neurons expressed HAP1. In contrast, HAP1 was completely lacking in TH-ir neurons. Our current study is the first to clarify that HAP1 is highly expressed in nociceptive/proprioceptive neurons but absent in light-touch-sensitive TH neurons, suggesting the potential importance of HAP1 in pain transduction and proprioception.

## Highlights

• This study is the first to examine HAP1-expression in dorsal root ganglia (DRG).
• HAP1 is highly co-expressed with the markers of nociceptive/proprioceptive neurons.
• HAP1 is completely lacking in the touch-sensitive DRG neurons.
• HAP1 may play an important role in modulating nociceptive/proprioceptive functions.
• It will be of great interest to clarify the pathophysiological role of HAP1 in DRG.

## 1 Introduction

Huntingtin-associated protein 1 (HAP1) is a cytoplasmic protein that is abundantly expressed in different regions of the brain and spinal cord (Li et al., 1996; Gutekunst et al., 1998; Fujinaga et al., 2004, 2007, 2009; Islam et al., 2012, 2017; Wroblewski et al., 2018; Chen et al., 2020). HAP1 is often localized to the stigmoid body (STB), a spherical-to oval-shaped, non-membranous neurocytoplasmic inclusion of granular to fuzzy texture with low-moderate electron density (Shinoda et al., 1992, 1993; Gutekunst et al., 1998; Fujinaga et al., 2009; Islam et al., 2012, 2017). Transfection of HAP1 cDNA into different cultured cells can also induce the development of STB (Li et al., 1998a; Takeshita et al., 2006; Fujinaga et al., 2007, 2011), and HAP1 is thus considered as a determinant marker for STB (Li et al., 1998a; Fujinaga et al., 2007; Islam et al., 2017; Wroblewski et al., 2018).

HAP1 was initially recognized as a polyglutamine (polyQ) length-dependent interactor of huntingtin (htt), the gene product responsible for Huntington’s disease (Li et al., 1995). STB/HAP1 can protect against apoptosis and cell death induced by htt with an expanded polyQ sequence (Li et al., 2003; Metzger et al., 2008; Liu et al., 2020). STB/HAP1 can also bind to a polyQ-expanded androgen receptor derived from spinal and bulbar muscular atrophy (SBMA), and over expression of HAP1 suppresses polyQ androgen receptor-induced apoptosis (Takeshita et al., 2006). STB/HAP1 can also interact with the causal agents of some other polyQ diseases, such as with Abelson helper integration site 1 in Joubert syndrome (Sheng et al., 2008), ataxin 3 in Machado-Joseph disease (Takeshita et al., 2011) and TATA binding protein in spinocerebellar ataxia type 17 (Prigge and Schmidt, 2007). In addition, STB/HAP1 is copiously expressed in the limbic-hypothalamic regions of brain and dorsal horn of spinal cord in normal rodents (Fujinaga et al., 2004; Takeshita et al., 2006, 2011; Islam et al., 2012, 2017; Wroblewski et al., 2018; Chen et al., 2020). Interestingly, these regions of the central nervous system are usually spared from neurodegeneration, whereas the regions lacking STB/HAP1 or with little expression such as neocortex, striatum, thalamus, cerebellum and spinal motoneurons are major targets in different neurodegenerative diseases (Fujinaga et al., 2004; Islam et al., 2017). Taken together, STB/HAP1 is thought to augment the threshold of vulnerability to neurodegenerative apoptosis, confer increased neuronal stability, and subsequently protect against cell death and apoptosis in several neurodegenerative diseases. This has been referred to as the “STB/HAP1 protection hypothesis” (Fujinaga et al., 2004; Metzger et al., 2008; Takeshita et al., 2006; Islam et al., 2017; Wroblewski et al., 2018).

In terms of physiological functions, several studies have reported that HAP1 can act as a mediator of feeding behaviors (Chan et al., 2002; Dragatsis et al., 2004; Sheng et al., 2006; Lin et al., 2010; Niu et al., 2011), modulate hypothalamic function for stress response (Chen et al., 2020) or play a vital role in early brain development (Sheng et al., 2008). In our own recent study, we showed that STB/HAP1 is highly expressed in the dorsal horn of the spinal cord, suggesting that STB/HAP1 may participate in modifications of certain sensory functions (Islam et al., 2017). It is well-known that cell bodies of the sensory neurons reside in the dorsal root ganglia (DRG) and in the trigeminal ganglia. These neurons have single process that diverges, dispatching one branch to the spinal cord/brain stem and another one to the periphery (Le Pichon and Chesler, 2014). DRG contains a heterogenous population of sensory neurons including neurons responsible for nociception, sensation of itch, thermoception, proprioception and touch sensation (Lallemend and Ernfors, 2012; McCoy et al., 2013; Pogorzala et al., 2013). It is possible that STB/HAP1 is involved in processing or modification of certain sensory functions in the DRG. In this context, it becomes important to clarify the expression of HAP1in the DRG and to examine its relationships with sensory neuron subpopulations. Substance P (SP) and calcitonin gene-related peptide (CGRP) are the two well-known markers for peptidergic nociceptors (Emery and Ernfors, 2018). Calbindin (CB) and nitric oxide synthase (NOS) are considered to play a vital role in the induction and transduction of nociception (Patil et al., 2006; Egea et al., 2012), whereas transient receptor potential vanilloid 1 (TRVP1) is the receptor for capsaicin, which has been shown to be heat sensitive (Caterina et al., 1997). Calretinin (CR) and parvalbumin (PV) are considered to play a vital role in proprioception (Ren et al., 1993; de Nooij et al., 2013; Medici and Shortland, 2015), whereas tyrosine hydroxylase (TH) is believed to modulate light-touch sensation (Brumovsky, 2016). These neurochemical markers show species or regional differences in percentage expression and distribution in DRG. Substance P is present in 10–30 % of the DRG neuronal populations, usually limited to small or medium sized sensory neurons (Lawson, 1992; Otsuka and Yoshioka, 1993). The percentage of CGRP-positive DRG neurons is about 30 % (Zwick et al., 2002). Approximately 14 % of DRG neurons are reported to be PV-positive and usually localized in large-diameter neurons, with a similar proportion for CB in small- to medium-sized neurons and around 10 % of medium- to large-sized neurons are CR-positive (Carr et al., 1989; Ren et al., 1993; Honda, 1995). On average 37 % of all DRG neurons express TRPV1 (Cho and Valtschanoff, 2008) and about 15–37 % of DRG neurons express TH (Usoskin et al., 2015). To date, however, analysis of HAP1 expression in the DRG and its neurochemical characterization in reference to sensory neuron subpopulations have not been conducted.

In the present study, we set out to clarify the expression and detailed distribution of HAP1 in the adult mouse DRG at different levels of the spinal cord (cervical to sacral). We also aimed to elucidate the immunohistochemical relationships of HAP1 with two principally different types of neuron that govern sensory information, the nociceptors that carry pain or thermal sensation and mechanoreceptors that carry touch sensation or proprioception.

## 2 Materials and methods

### 2.1 Animals and ethical approval

Adult male C57BL/6 J mice (8-week-old) were purchased from Japan SLC Inc., (Shizuoka, Japan) for the current study. The animals were kept in groups (3–4 mice) at 22−24 °C temperature with a 12 to 12-h light dark cycle (lights on 08:00−20:00) and provided water and food *ad libitum*.

Experimental protocols used in this study were approved by the Yamaguchi University School of Medicine Committee on the Ethics of Animal Experimentation and carried out according to the guidelines for Animal Research of the Government of Japan (Law No. 105, Notification No. 6). A total of 30 male mice were used for the current study. Among them 6 mice were used for Western blotting and 24 mice for immunohistochemistry (6 mice for immunoperoxidase staining and 18 mice for immunofluorescence staining). All efforts were employed to reduce the number of mice used and their suffering.

### 2.2 Primary antibodies

**Fig. 1:**
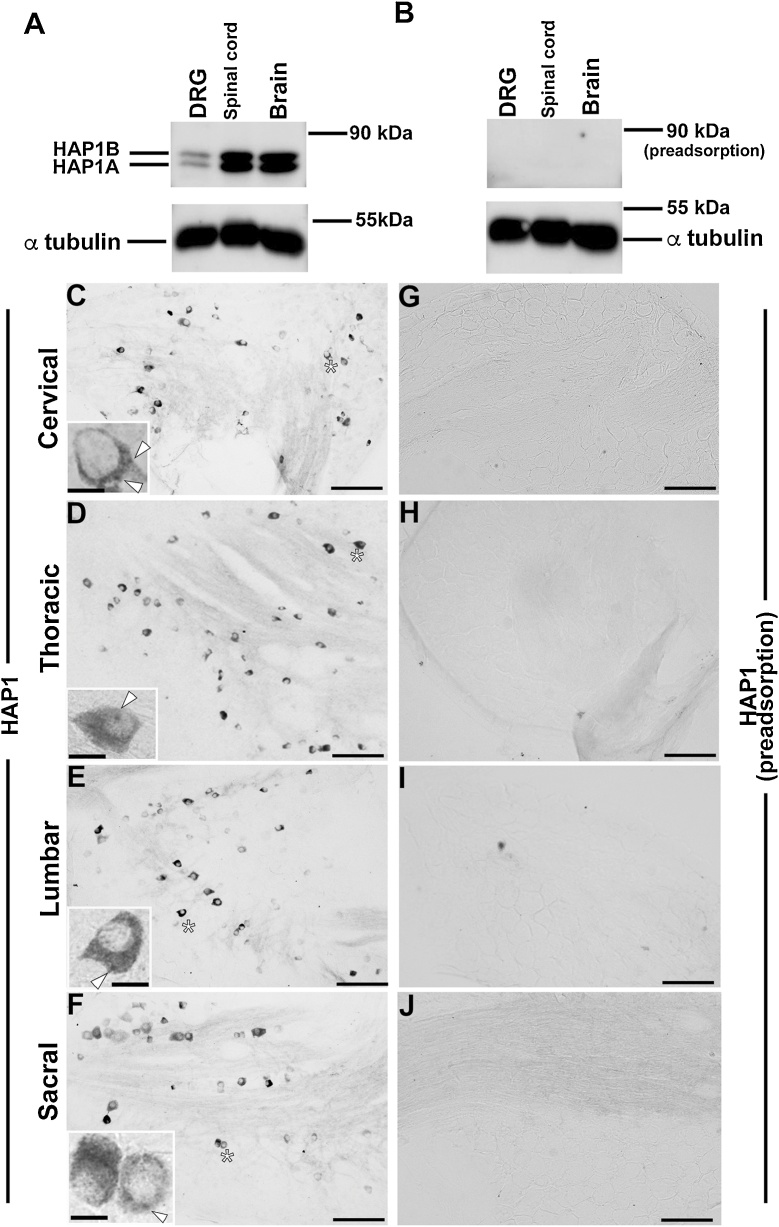
Western blotting and immunohistochemistry for huntingtin-associated protein 1 (HAP1). (A) Western blot analysis using lysate from the dorsal root ganglion (DRG), spinal cord and whole brain showing bands of approximately 85 KDa for HAP1B and 75 KDa for HAP1A. (B) Pre-adsorption of the anti-HAP1 antibody with a blocking peptide against HAP1 antibody resulted in disappearance of the HAP1-positive bands. α tubulin was used for loading control. (C–F) Immunohistochemistry showing the presence of HAP1-immunoreactive (ir) cells in the DRG of different spinal level. (G–J) Preincubation with a blocking peptide against the anti-HAP1 antibody eliminated the HAP1-immunoreactivity. Arrowheads indicate the HAP1-ir stigmoid body in the cytoplasm. Scale bar = 100 μm in C–J and 10 μm in insets of. C–F.

The details of the primary antibodies used in the current study are listed in Table 1, all of which are commercially available. The characterization of these primary antibodies was clarified in our previous studies or earlier by others (Table 1). In addition, the characterization of the anti-HAP1 primary antibody was also determined in the present study using the pre-adsorption test (Fig. 1).

**Table 1:** List of primary antibodies used in the present study.

Antibody	Immunogen	Code	Host /clonality	Source	Dilution	References
HAP1 (R19)	Rat HAP1 C-terminus	Cat# sc-8770, RRID: AB_647322	Goat polyclonal	Santa Cruz Biotechnology, Santa Cruz, CA	1: 10,000	Islam et al., 2012, 2017
HAP1 (mouse)	Mouse HAP1 C-terminus	Cat# EB07787, RRID: AB_2116122	Goat polyclonal	Everest Biotech Ltd, Oxfordshire, UK	1:10,000	Characterized in the present study
CB	Recombinant rat calbindin d-28 K	Cat# CB38, RRID: AB_2721225	Rabbit polyclonal	Swant, Marly, Switzerland	1:5,000	Graïc et al., 2018
CGRP	CGRP-KLH (rat)	Cat# C8198, RRID: AB_259091	Rabbit polyclonal	Sigma-Aldrich, St. Louis, MO	1:1,000	Russo et al., 2013
CR	Recombinant human calretinin	Cat# CR6797, RRID: AB_2619710	Rabbit polyclonal	Swant, Marly, Switzerland	1:1,000	Ch’ng et al., 2019
Iba1	Synthetic peptide corresponding to the C-terminus of Iba1	Cat# 019−19741, RRID: AB_839504	Rabbit polyclonal	Wako, Osaka, Japan	1:1,000	Yamanaka et al., 2011
NeuN	Synthetic peptide of Human NeuN aa 1−100	Cat# ab177487, RRID: AB_2532109	Rabbit monoclonal	Abcam, Cambridge, UK	1:5,000	Saito et al., 2018
NOS	C-terminus synthetic peptide of human nNOS coupled to KLH	Cat# 24287, RRID: AB_572256	Rabbit polyclonal	Immunostar, Hudson, WI, USA	1:1,000	Bilella et al., 2016
PV	Recombinant rat parvalbumin	Cat# PV27, RRID: AB_2631173	Rabbit polyclonal	Swant, Marly, Switzerland	1:1,000	Ordás et al., 2019
SP	Synthetic SP coupled to KLH with carbodiimide	Cat# 20064, RRID: AB_572266	Rabbit polyclonal	Immunostar, Hudson, WI, USA	1:1,000	Kestell et al., 2015
TH	Denatured TH from rat pheochromocytoma	Cat# AB152, RRID: AB_390204	Rabbit polyclonal	Millipore, Billerica, MA, USA	1:1,000	Rosinger et al., 2019
TRPV1	Peptide corresponds to absolute C- terminus of mouse TRPV1 sequence	Cat# RA14113, RRID: AB_2194034	Rabbit polyclonal	Neuromics, Edina, MN, USA	1:1,000	Ritter and Southard-Smith, 2017
α tubulin	Microtubule derived from chicken embryonic brain	Cat# T6199, RRID: AB_477583	Mouse monoclonal	Sigma-Aldrich, St. Louis, MO	1:20,000	König et al., 2014

HAP1, huntingtin-associated protein 1; CB, calbindin; CGRP, calcitonin gene related peptide; CR, calretinin; Iba1, ionized calcium-binding adapter molecule 1; KLH, keyhole limpet hemocyanin; NeuN, neuronal nuclei; NOS, nitric oxide synthase; PV, parvalbumin; SP, substance P; TH, tyrosine hydroxylase; TRPV1, transient receptor potential vanilloid 1.

### 2.3 Western blotting

Western blotting was conducted as described in our earlier studies (Islam et al., 2017, 2020). In brief, immediately after obtaining the whole brain, spinal cord or DRGs (cervical, thoracic, lumbar and sacral combined) were homogenized in T-PER™ tissue protein extraction reagent (78,510; Thermo Scientific, Rockford, IL, USA) containing 5μl/mL of protease inhibitor (P8340; Sigma-Aldrich). Pierce™ BCA Protein Assay Kit (23,227; Thermo Scientific, Waltham, MA, USA) was used to measure the protein concentration in each sample. An equal amount of each protein was loaded and separated by 7.5 % SDS-polyacrylamide gel electrophoresis and then transferred onto a polyvinylidene difluoride membrane by wet transfer apparatus. After blocking for 1 h at 20 °C with 5% skim milk (190–12865; FUJIFILM Wako Pure Chemical Corporation, Osaka, Japan) in Tris-buffered saline with 0.1 % Tween (TBST), the membrane was incubated overnight at 4 °C in blocking solution with goat polyclonal anti-HAP1 (1:10,000) or mouse monoclonal anti-α tubulin (1:200,000) antibodies (Table 1). The diluted antibody was incubated overnight at 4 °C with a specific blocking peptide for the HAP1 pre-adsorption test. After three washes, the membrane was incubated with horseradish peroxidase-linked anti-goat (1:5,000; SC-3851, Santa Cruz Biotechnology) or anti-mouse IgG (1:20,000; GE Healthcare, Buckinghamshire, UK) antibody for 2 h at 20 °C. Finally, after washing three times in TBST, protein bands were examined using enhanced chemiluminescence reagents (ECL select, GE Healthcare) and images were captured with Amersham Imager 600 (GE Healthcare).

### 2.4 Tissue preparation for immunohistochemistry

Mice were transcardially perfused with 4% paraformaldehyde in 0.1 M phosphate buffer (PB; pH 7.4) under anesthesia with pentobarbital sodium (60−80 mg/kg, intraperitoneal injection). The DRGs (cervical to sacral) were extracted by laminectomy (Sleigh et al., 2016), post-fixed for overnight in the same fixative used for perfusion and then transferred to 0.1 M PB containing 30 % sucrose solution for several days. Finally, the DRGs were frozen in powdered dry ice and then sectioned at a thickness of 40 μm on a cryostat.

### 2.5 Single immunoperoxidase histochemical staining

Single immunoperoxidase immunohistochemistry was performed as described in our previous studies (Islam et al., 2012, 2017, 2020). In brief, free floating sections of DRG (cervical to sacral) were blocked with 10 % normal donkey serum (NDS; S30−100ML, Millipore, Temecula, CA, USA) containing 0.3 % Triton X-100 at room temperature for 2 h, pretreated with 1.5 % hydrogen peroxide and 50 % methanol at 4 °C for 30 min and then incubated with primary antibodies to HAP1 (1: 10,000) at 20 °C for 5 d. For the pre-adsorption test, the diluted primary antibody was incubated at 4 °C overnight with a specific blocking peptide against HAP1 antibody. Then, after washing three times the sections were incubated at 20 °C for 2 h with biotinylated donkey anti-goat secondary antibody (AP180B, Millipore; 1:1,000 dilution) followed by incubation at 20 °C for 2 h with peroxidase-conjugated streptavidin (1:1,000 dilution; Dako, Glostrup, Denmark). After washing three times with 0.05 M Tris−HCl buffer (pH 7.6), the sections were processed for nickel-enhanced diaminobenzidine (DAB) reaction at 4 °C for 10−20 min with a mixture of 0.02 % 3, 3^/^ DAB; (Dojinbo Laboratories, Kumamoto, Japan) and 0.6 % nickel ammonium sulfate (Sigma-Aldrich, Tokyo, Japan) in 0.05 M Tris−HCl buffer containing 0.0008 % H_2_O_2_. Finally, the sections were mounted on glass slides, air-dried for 30 min, dehydrated using graded series of alcohol and Xylene and lastly embedded with Entellan New (Millipore).

### 2.6 Double-label immunofluorescence histochemical staining

Double-label immunofluorescence immunohistochemistry was carried out as described in our previous reports (Jahan et al., 2015; Islam et al., 2017). In brief, free floating sections of DRG (cervical to sacral) were blocked with 10 % NDS containing 0.3 % Triton X-100 at 4 °C for 2−3 h and incubated with goat anti-HAP1 (1:10,000) antibody in combination with a rabbit anti-SP (1:1,000), rabbit anti-CGRP (1:1,000), rabbit anti-CB (1:20,000), rabbit anti-NOS (1:1000), rabbit anti-TRPV1 (1:1000), rabbit anti-CR (1:1,000), rabbit anti-PV (1:1,000), rabbit anti-TH (1: 1,000), rabbit anti-ionized calcium-binding adapter molecule 1 (Iba1; 1: 1,000) or rabbit anti-neuronal nuclei (NeuN; 1: 1,000) antibody at 20 °C for 5 d (Table 1 for detail about primary antibodies). After washing three times the sections were incubated with a mixture of Alexa Fluor 594-conjugated donkey anti-goat IgG (A11058, AB_2534105, Invitrogen, Eugene, OR, USA; 1:1,000) and Alexa Fluor 488-conjugated donkey anti-rabbit IgG (A32790, AB_2762833, Invitrogen, Rockford, IL, USA; 1:1,000) secondary antibodies at 20 °C for 2−3 h. After washing three times with PBS, the sections were then mounted on glass slides, air-dried for 30 min and finally embedded with Fluoromount/Plus (K048, Diagonostic Biosystems, Pleasanton, CA, USA).

### 2.7 Photomicrographs

For immunoperoxidase staining images, a color digital Lumenera USB 2.0 camera (Lumenera Corporation, Ottawa, Canada) equipped with an Eclipse E80i photomicroscope (Nikon) was used to capture photomicrographs. For immunofluorescence staining images, a laser-scanning microscope (LSM510; Carl Zeiss, Jena, Germany) was used to obtain a single optical sections (1024 × 1024 pixels). Images were then transferred onto Adobe Photoshop Elements 2018 (Adobe Systems, Inc., San Jose, CA, USA) where only image contrast and brightness were modified (applied to the whole image) to achieve better quality images.

### 2.8 Tissue analyses and cell counting

For tissue analyses, immunofluorescence images were taken using 20 x objective and transferred into imageJ software (NIH, Bethesda, MD, USA). Cells with a clearly visible nucleus were evaluated. Immunoreactive cell was defined as positive when it was 1.5–2 standard deviations above the mean background fluorescence of unlabeled cell (Kestell et al., 2015). Profile counting of HAP1-immunoreactive (ir) cells was performed by comparing the total number of nucleated neurons stained by NeuN with the total number of HAP1-positive neurons. For size distribution analysis, DRG neurons were divided into three size groups: small, <300 μm^2^; medium‐sized, 300–700 μm^2^ and large, >700 μm^2^ (Ruscheweyh et al., 2007). Profile counting, quantification of size distribution and evaluation of co-expression ratios for HAP1/ markers or markers / HAP1 were performed on 4th - 5th cervical (C4 - C5), 4th - 5th thoracic (T4 -T5), 3rd - 4th lumbar (L3 - L4), and 1st - 2nd sacral (S1- S2) regions of the DRG using four sections from each level of one mouse. Two pairs of sections (never closure than 80 μm to next pair) were randomly chosen from each two ganglia per spinal level. At least 100 HAP1-ir neurons were counted from one mouse. A total of 6 mice were used for each quantification. Values were shown as mean ± SEM (n = 6).

Co-expression ratios for HAP1/nociceptive markers or nociceptive markers / HAP1 (Table 2) were calculated from the actual number of HAP1-ir cells and nociceptive markers-ir cells, and from those double-stained for HAP1 and nociceptive markers following the counting procedure described in our previous study (Nagano and Shinoda, 1994). The immunopositive cells was counted on C4 - C5, T4 -T5, L3 - L4 and S1- S2 regions of the DRG using four sections from each level for every combination of HAP1 and a particular marker (sixteen sections from one mouse for each combination of HAP1 and one particular marker). The co-expression ratios for HAP1/mechanoreceptive markers or mechanoreceptive markers /HAP1 (Table 3) were also estimated accordingly.

**Table 2:** Co-expression ratios of HAP1/nociceptive markers and nociceptive markers/HAP1 in the different levels of mouse DRG.

Relationship of HAP1 with different markers (ratio %)		Cervical	Thoracic	Lumbar	Sacral
SP	HAP1/SP	71.12 ± 4.3	75.91 ± 6.3	71.91 ± 9.8	72.91 ± 4.1
	SP/HAP1	55.43 ± 5.5	56.59 ± 8.1	60.12 ± 7.4	62.27 ± 7.6
CGRP	HAP1/CGRP	84.71 ± 6.2	82.76 ± 9.4	85.76 ± 3.3	78.34 ± 5.2
	CGRP/HAP1	68.64 ± 8.3	74.21 ± 6.2	75.31 ± 9.1	69.75 ± 7.5
CB	HAP1/CB	80.39 ± 6.1	82.61 ± 3.2	91.83 ± 4.2	89.32 ± 2.8
	CB/HAP1	58.56 ± 9.1	63.33 ± 8.5	67.78 ± 7.1	71.23 ± 7.3
NOS	HAP1/NOS	75.67 ± 8.2	81.25 ± 7.5	87.43 ± 6.2	85.33 ± 4.1
	NOS/HAP1	35.56 ± 5.2	40.64 ± 2.8	44.75 ± 9.1	39.14 ± 7.2
TRPV1	HAP1/TRPV1	70.36 ± 6.9	78.57 ± 2.5	79.39 ± 4.1	81.48 ± 5.6
	TRPV1/HAP1	52.34 ± 7.4	57.12 ± 8.3	61.34 ± 9.1	56.89 ± 3.5

Values represent the mean ± SEM (n = 6). CB, calbindin; CGRP, calcitonin gene-related peptide; NOS, nitric oxide synthetase; SP, substance P; TRPV1, transient receptor potential vanilloid 1.

**Table 3:** Co-expression ratios of HAP1/mechanoreceptive markers and mechanoreceptive markers/HAP1 in the different levels of mouse DRG.

Relationship of HAP1 with different markers (ratio %)		Cervical	Thoracic	Lumbar	Sacral
CR	HAP1/CR	63.12 ± 9.2	69.12 ± 7.1	78.23 ± 3.2	71.54 ± 5.7
	CR/HAP1	9.63 ± 6.3	10.34 ± 3.9	12.62 ± 7.9	14.18 ± 8.2
PV	HAP1/PV	84.71 ± 6.2	88.57 ± 5.9	89.13 ± 7.3	83.15 ± 9.8
	PV/HAP1	76.38 ± 7.1	74.21 ± 6.2	79.41 ± 7.8	75.51 ± 8.2
TH	HAP1/TH	0	0	0	0
	TH/HAP1	0	0	0	0

Values represent the mean ± SEM (n = 6). CR, calretinin; PV, parvalbumin; TH, tyrosine hydroxylase.

### 2.9 Statistical analysis

One-way- analysis of variance (ANOVA) was performed to reveal any difference among different spinal levels in the cell profile and size distribution of HAP1-ir neurons in the DRG. A two-sided P-value of <0.05 was considered statistically significant. A software package SPSS version 22 for Windows (SPSS Inc., Chicago, IL, USA) was used for statistical analyses.

## 3 Results

### 3.1 Expression of HAP1-immunoreactivity in the DRG

The expression of HAP1 in the DRG was determined by both Western blotting and immunohistochemistry. In Western blotting, both isoforms of HAP1 (approximately 85 and 75 kDa for HAP1B and HAP1A, respectively) were detected in the mouse DRG, although the expression was substantially lower than that in the brain or spinal cord (Fig. 1A). HAP1-ir bands were completely eliminated in the pre-adsorption test (Fig. 1B). In immunohistochemistry, a number of HAP1-ir cells were detected in the DRG throughout the spinal levels (cervical to sacral) (Fig. 1C-F). The distribution pattern of HAP1-ir cells was generally similar at all the spinal levels of DRG (Fig. 1C-F). In pre-adsorption test, HAP1-immunoreactivity was eliminated in DRG of all spinal levels (Fig. 1G-J). Some HAP1-ir cells had dot-like STBs in their cytoplasm, while other HAP1-ir cells had only diffuse staining in their cytoplasm with undetectable STBs (Fig. 1C-F). Usually, most HAP1-ir cells express both isoforms of HAP1 and cells with comparatively more HAP1A induce the development of STB in their cytoplasm while those with comparatively more HAP1B display diffuse HAP1-immunoreactivity in their cytoplasm (Fujinaga et al., 2007; Islam et al., 2017; Wroblewski et al., 2018). It is thus somewhat difficult to discern the staining of HAP1A from that of HAP1B in immunohistochemistry, though the antibody to HAP1 used in the current study can detect both isoforms of HAP1 in Western blotting.

**Fig. 2:**
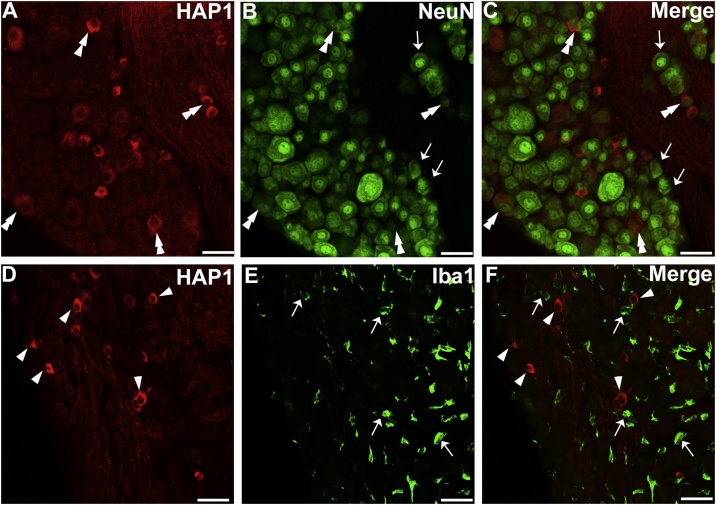
Double-label immunofluorescence immunohistochemistry for HAP1 with NeuN or Iba1. Photomicrograph showing double-label immunofluorescence staining of HAP1 and NeuN (A-C) or Iba1 (D-F) in the thoracic DRG. Arrows indicate cells for single-positive for HAP1. Arrowheads indicate cells single-positive for NeuN or Iba1. Double arrowheads indicate the cells positive for both HAP1 and NeuN. Scale bar = 50 μm in A-F.

To characterize the type of HAP1-ir cells, double-label immunostainings for HAP1 and Iba1 (marker for macrophages) or NeuN (neuronal marker) were performed (Fig. 2). Almost all the HAP1-ir cells exhibited clear NeuN-immunoreactivity (Fig. 2A-C) but were negative for Iba1 (Fig. 2D-F), indicating that HAP1-ir cells in the DRG showed attributes of neurons but not of macrophages.

**Fig. 3:**
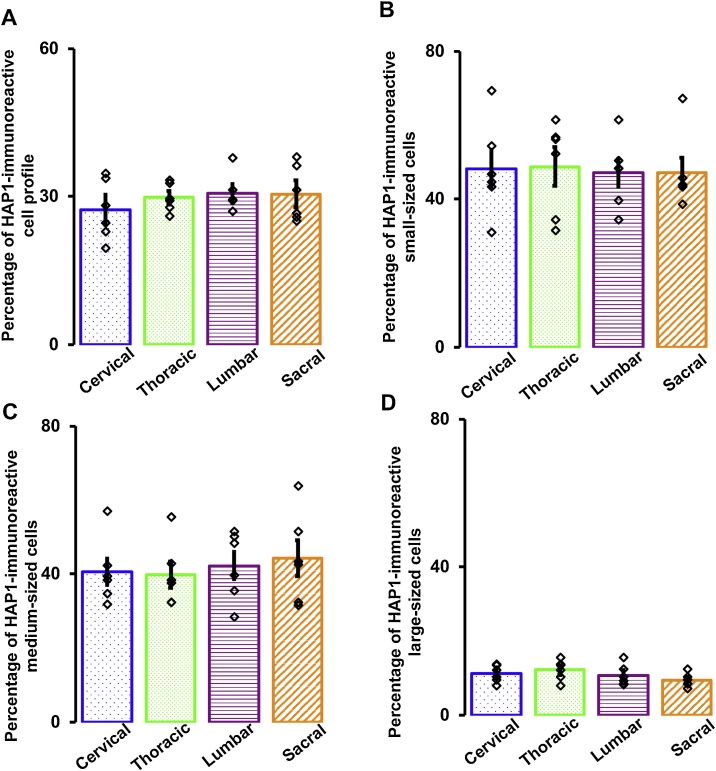
Analysis of cell profile and size distribution of HAP1-ir neurons in the DRG. Bar graphs showing (A) percentage of HAP1-ir neuron profile and (B-D) size distribution of HAP1-ir neurons in the DRG of different spinal level. Values represent the mean ± SEM (n = 6).

In our current study, the percentage of HAP1-ir DRG neurons in adult mice ranged between 28–31% in the different spinal (cervical to sacral) levels (Fig. 3A). However, we did not find any significant difference in the number of HAP1-ir DRG neurons among the spinal levels (P = 0.991, one-way ANOVA). Next, we examined the size distribution of HAP1-ir DRG neurons (Fig. 3B-D). In our current study, the HAP1-immunoreactivity was relatively more in the small cells (ranging between 47–58%) and medium cells (ranging between 40–44%) than that in the large cells (ranging between 9–11%). For the size distribution of HAP1-ir DRG neurons, we did not find any significant difference among the spinal levels as well (small cells, P = 0.991; medium cells, P = 0.869; large cells, P = 0.272; one-way ANOVA).

### 3.2 Immunohistochemical relationships of HAP1 with nociceptors

**Fig. 4:**
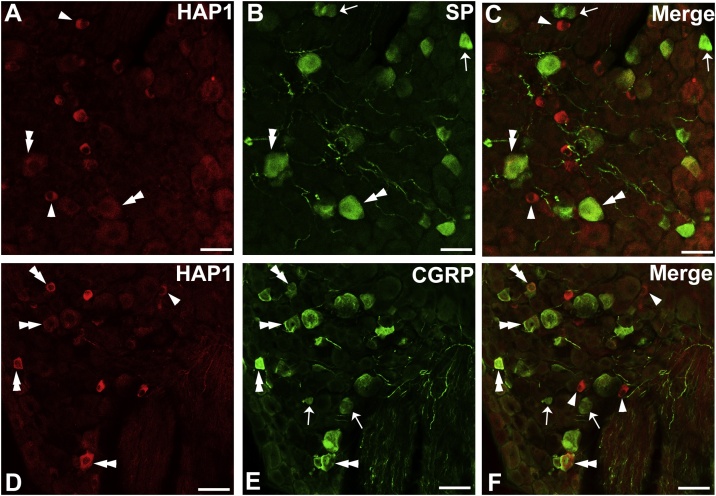
Double-label immunofluorescence immunohistochemistry for HAP1 with SP or CGRP. Photomicrograph showing double-label immunofluorescence staining of HAP1 and SP (A-C) or CGRP (D-F) in the thoracic DRG. Arrows indicate cells for single-positive for HAP1. Arrowheads indicate cells single-positive for SP or CGRP. Double arrowheads indicate the cells positive for both HAP1 and SP or CGRP. Scale bar = 50 μm in A-F.

To examine the morphological relationships between HAP1 and nociceptive neurons in the DRG, we first performed double-label immunofluorescence staining for HAP1 with SP and CGRP. Cell counting indicated that the co-expression ratio of HAP1 in SP-ir cells was 71–75 % and that of SP in HAP1-ir cells was 55–62 % (Fig. 4A–C; Table 2). Cell counting also revealed that the co-expression ratio of HAP1 in CGRP-ir cells was 78–84 % and that of CGRP in HAP1-ir cells was 68–75 % (Fig. 4D–F; Table 2).

**Fig. 5:**
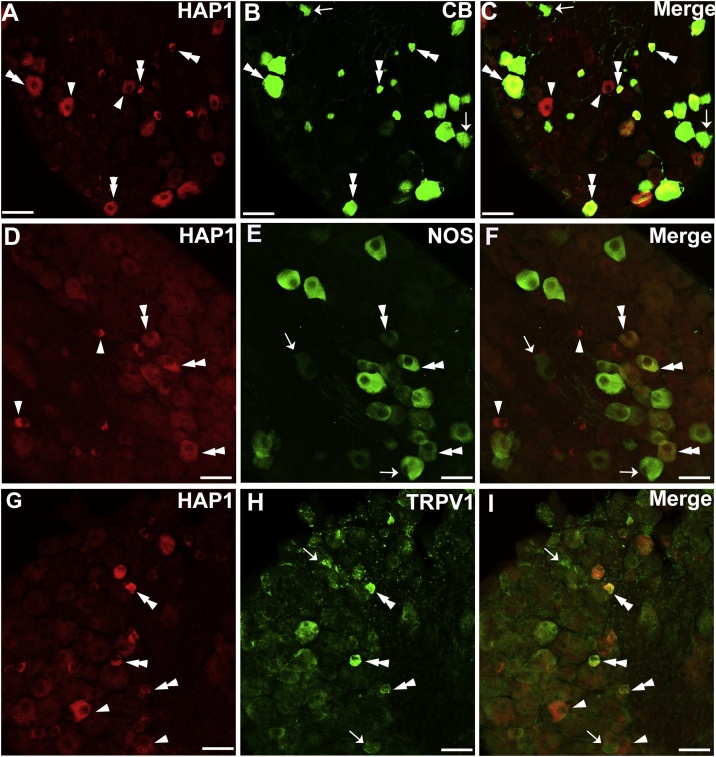
Double-label immunofluorescence immunohistochemistry for HAP1 with CB, NOS, or TRPV1. Photomicrograph showing double-label immunofluorescence staining of HAP1 and CB (A-C), NOS (D-F) or TRPV1 (G-I) in the thoracic DRG. Arrows indicate cells for single-positive for HAP1. Arrowheads indicate cells single-positive for CB, NOS, or TRPV1. Double arrowheads indicate the cells positive for both HAP1 and CB, NOS or TRPV1. Scale bar = 50 μm in A-I.

Next, double-label immunofluorescence staining was performed for HAP1 with CB, NOS and TRPV1. Our cell counting showed that the co-expression ratio of HAP1 in CB-ir cells was 80–91 % and that of CB in HAP1-ir cells was 58–71 % (Fig. 5A–C; Table 2). On the other hand, the co-expression ratio of HAP1 in NOS-ir cells was 75–85 % and that of NOS in HAP1-ir cells was 35–45 % (Fig. 5D–F; Table 2). Our cell counting also revealed that the co-expression ratio of HAP1 in TRPV1-ir cells was approximately 70–81 % and that of TRPV1 in HAP1-ir cells was 52–61 % (Fig. 5G–I; Table 2).

### 3.3 Immunohistochemical relationships of HAP1 with mechanoreceptors

**Fig. 6:**
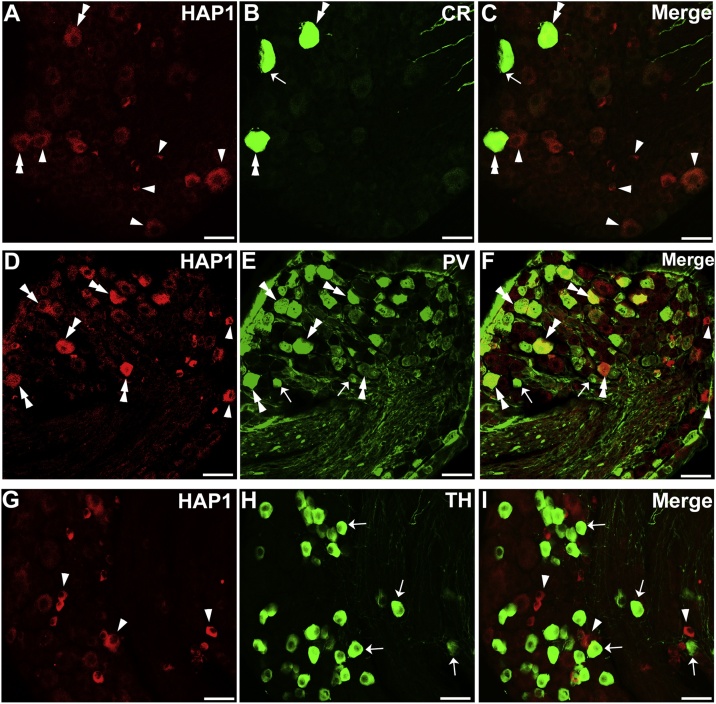
Double-label immunofluorescence immunohistochemistry for HAP1 with CR, PV or TH. Photomicrograph showing double-label immunofluorescence staining of HAP1 and CR (A-C), PV (D-F) or TH (G-I) in the thoracic DRG. Arrows indicate cells single-positive for HAP1. Arrowheads indicate cells single-positive for CR, PV, or TH. Double arrowheads indicate the cells positive for both HAP1 and CR or PV. Scale bar = 50 μm in A-L.

To examine the morphological relationships between HAP1 and mechanoreceptive neurons in DRG, we performed double-label immunofluorescence staining for HAP1 with CR, PV, and TH. Cell counting indicated that the co-expression ratio of HAP1 in CR-ir cells was 63–71 % and that of CR in HAP1-ir cells was 9–14 % (Fig. 6A–C; Table 3), whereas the co-expression ratio of HAP1 in PV-ir cells was 83–89 % and that of PV in HAP1-ir cells was 74–76 % (Fig. 6D–F; Table 3). Intriguingly, in contrast, our current double-immunofluorescence results revealed that HAP1 was never detected in TH-expressing neurons of DRG (Fig. 6G–I; Table 3).

## 4 Discussion

The current study employing Western blot and immunohistochemistry is the first to determine the expression and distribution of HAP1in the DRG throughout the cervical to sacral regions in adult male mouse. The present study is also the first to characterize HAP1 immunoreaction in relation to the sensory neuron subpopulations in the mouse DRG. Although the expression of HAP1 has been analyzed in the central nervous system previously in a number of studies (Li et al., 1996; Gutekunst et al., 1998; Dragatsis et al., 2004; Sheng et al., 2006, 2008; Lin et al., 2010; Niu et al., 2011; Fujinaga et al., 2004, 2007, 2009; Islam et al., 2012, 2017; Wroblewski et al., 2018; Chen et al., 2020), its expression, cellular localization, regional distribution, and neurochemical characterization have never been reported in the peripheral nervous system. Our Western blotting results clearly enunciated that both HAP1A and HAP1B isoforms were expressed in mouse DRG, and our immunohistochemical results demonstrated the presence of HAP1-immunoreactions in the cytoplasm of DRG neurons, as previously observed in the brain and spinal cord (Li et al., 1998a; Gutekunst et al., 1998; Fujinaga et al., 2009; Islam et al., 2017). The present study shows that HAP1-immunoreactivity is present not only in the brain and spinal cord of the central nervous system but also in the DRG of the peripheral nervous system.

**Fig. 7:**
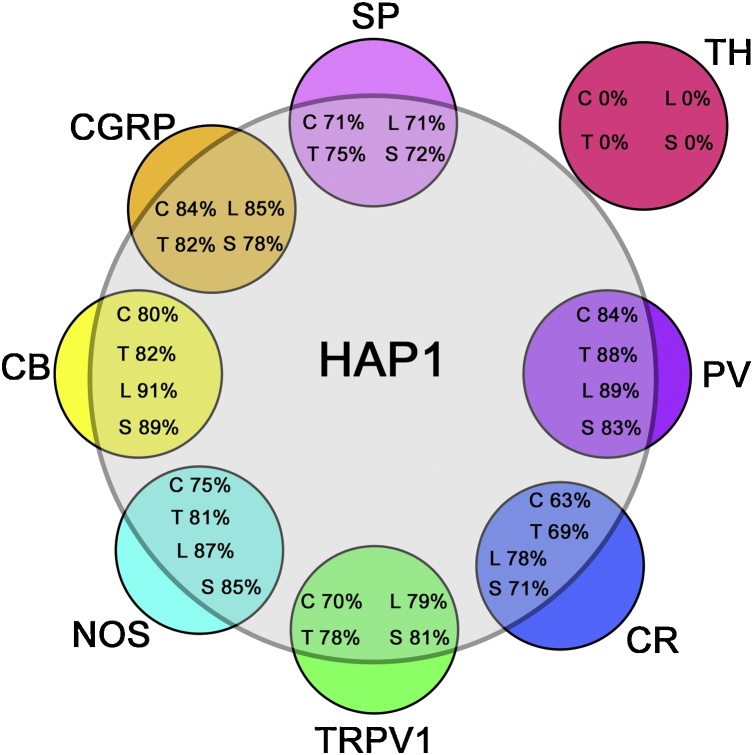
Pseudo Venn-diagram indicating the relative extent to which nociceptive or mechanoreceptive markers express HAP1 in DRG. The extent to which nociceptive or mechanoreceptive markers themselves are co-expressed in DRG is not indicated. The sizes of the circles depicting the nociceptive or mechanoreceptive markers is not representative of the relative number of the neurons that express those markers.

Somatic sensory neurons in DRG transmit diverse sensory information from the skin, bones, muscles, and visceral organs (Liu and Ma, 2011). These includes i) nociceptvie lineage that responds to pain, thermal and itch sensation, and ii) mechanoreceptors that respond to body positions (proprioception) or touch and vibration (Basbaum et al., 2009; Lallemend and Ernfors, 2012; McCoy et al., 2013; Pogorzala et al., 2013; Bartesaghi et al., 2019). The most striking finding in the current study, however, is that a high percentage of the nociceptive and proprioceptive neurons express HAP1-immunoreactivity, while the light-touch-sensitive neurons are specifically devoid of HAP1-immunoreactivity across the different spinal levels of the DRG (summarize in Fig. 7). This probably reflects HAP1′s involvement in modulating somatosensory and viscerosensory information under particular physiological conditions.

### 4.1 Possible involvement of HAP1 in pain and thermal sensation of nociceptive lineage

Nociceptive sensation is important for preserving the functional integrity of the body (Bartesaghi et al., 2019). Nociceptive DRG neurons are mainly classified into nonpeptidergic and peptidergic groups. Peptidergic nociceptors express neuropeptides, usually CGRP or SP, while nonpeptidergic unmyelinated nociceptors do not express neuropeptides but bind to isolectin B4 (Julius and Basbaum, 2001; Dubin and Patapoutian, 2010; Kestell et al., 2015). Other than these two conventional nociceptors, there are some DRG nociceptor subpopulations that neither express neuropeptides nor bind to isolectin B4, instead express vesicular glutamate transporter (VGLUT) 2 (Morris et al., 2005; Lagerström et al., 2010) or TRPV1 (Bráz and Basbaum, 2010). However, it has been shown that VGLUT2 is expressed virtually by all CGRP or IB4-ir neurons in mouse DRG (Brumovsky et al., 2007). CGRP is mainly expressed in small-medium sized neurons of the DRG. Most of the CGRP-positive DRG neurons also contain SP. The TRPV1 is a ligand-gated cation channel that is activated by noxious heat, capsaicin (Caterina et al., 1997; Kitamura et al., 2018). TRPV1 activation leads to the release of CGRP or SP from nerve terminals. However, there are some large-sized CGRP-expressing DRG neurons that contain neither SP- nor TRPV1-immunoreactions (Kestell et al., 2015). In the current study, we found that about 70 % of SP, 75 % of CGRP and 80 % of TRPV1 neurons expressed HAP1. Abundant expression of HAP1-immunoreactivity in the SP-, CGRP- or in TRPV1-ir neurons might imply a vital role of HAP1 in modulation of pain and thermoreceptive functions. Detailed physiological and morphological experiments are needed in future to elucidate the effects of HAP1 on the nociceptive functions.

It has been reported that pain transmission involves a Ca^2+^ regulating system that consists of the entry of Ca^2+^ through calcium channels and intracellular Ca^2+^ binding activity by CB in the particular nociceptive neurons of the DRG (Burnstock, 2006, 2007; Zeng et al., 2013; Zhang et al., 2014). In addition, nitric oxide plays a vital role in the transduction of the pain message (Patil et al., 2006). It is important to note that the current immunostaining for HAP1 and CB or NOS has provided very intriguing data that more than 80 % of CB-ir neurons or NOS-ir neurons express HAP1. This suggests that HAP1 probably plays an important role in the processing and transmission of the pain message. Future studies should include a marker for itch sensation to reveal the possible relationship of HAP1 with itch-sensitive sensory neurons of nociceptive lineage. It has been reported that VGLUT2, a potential marker for itch, overlaps with the TRPV1 subpopulation (Lagerström et al., 2010). Mas-related G-protein-coupled receptor (MRGPR) D or MRGPRA3 can also be used as markers for itch afferents (Le Pichon and Chesler, 2014). The expression of HAP1 in isolectin B4-positive nonpeptidergic DRG neurons, which convey mechanical nociceptive stimuli (Scherrer et al., 2009), will also need to be clarified in future studies.

### 4.2 Possible involvement of HAP1 in proprioception and touch sensation of mechanoreceptive linage

Proprioceptive sensory neurons convey information about body position and movement from peripheral receptors located in tendons, muscles or joints (Delhaye et al., 2018). Usually proprioceptive sensory neurons in the DRG have been recognized by their typically large neuron diameter (Lawson, 2002). Calcium-binding protein PV has been used as a marker of proprioceptive sensory neurons (Honda, 1995; de Nooij et al., 2013). It has been reported that PV is co-expressed with neurotrophin-3 and tyrosine receptor kinase C proteins, which are associated with the development of proprioceptive receptors and their primary afferent neurons (Ernfors et al., 1994). Furthermore, it has been mentioned that about 90 % of PV-ir DRG neurons are proprioceptors (de Nooij et al., 2013; Wralters et al., 2019). In addition, another calcium-binding protein CR is also regarded as a potential marker for proprioceptors, although it is expressed in a very small sub-population (10 %) of medium-large-sized DRG neurons (Ren et al., 1993). Another remarkable finding of our present study is that a large number of CR- and PV-ir neurons prominently express HAP1-immunoreactivity in the DRG, strongly suggesting that HAP1 plays an important role in modification of proprioceptive functions.

Touch sensation is important for social contacts, apprenticeship and sexuality. Innocuous touch is detected by the low-threshold mechanoreceptors (LTMRs) which are localized in the various layers of the skin (Roudaut et al., 2012). Cell bodies of LTMRs reside in the DRG and have been identified by their small neuron diameter (Li et al., 2011; Zimmerman et al., 2014). Based on the action potential conduction velocities, LTMRs are classified as Aβ, Aδ, or C. Aδ-LTMRs and Aβ-LTMRs are lightly and heavily myelinated, exhibiting intermediate and rapid conduction velocities respectively (Li et al., 2011), while C-LTMRs are unmyelinated and have the slowest conduction velocities. However, the number of C-LTMRs are 3–4 times more numerous than A-LTMRs (Abraira and Ginty, 2013), C-LTMRs generally transmit hair deflection, light-touch and cooling sensation (Brumovsky, 2016). C-LTMRs are also involved in modulating gentle and affective touch (Abraira and Ginty, 2013), which has been confirmed by examining the role of the T-type calcium channel Cav3.2 or the TAFA chemokine like family member 4, both highly co-localized with TH in C-LTMRs (Delfini et al., 2013; François et al., 2015). It has been reported that all C-LTMRs innervating hairy skin as longitudinal lanceolate nerve endings express TH (Li et al., 2011; Brumovsky, 2016). Moreover, TH-positive DRG neurons are a molecularly unique population of nonpeptidergic, small-diameter sensory neurons (Li et al., 2011). Taken together, TH is considered as a potential marker for gentle- or light-touch-sensitive DRG neurons (Brumovsky, 2016). However, TH-expressing DRG neurons may also participate in non-visceral pain transduction (Brumovsky, 2016). Our current results provide intriguing evidence that HAP1 never co-localize with TH in DRG. Although we need to analyze the immunohistochemical relationships of HAP1 with the markers of A-LTMRs in future, our present data suggest that HAP1 may not play an important role in modulating light-touch-sensation in a physiological sense. Instead, lack of HAP1-expression in light-touch-sensitive TH neurons indicates the vulnerability of these neurons to certain stresses, as described later.

### 4.3 Absence of HAP1 might indicate the vulnerability of light-touch-sensitive neurons to certain stresses

In the present study, the most striking data from a neuropathological viewpoint is that TH neurons in the DRG are devoid of HAP1-immunoreactivity. According to the STB/HAP1 protection hypothesis, lack of HAP1-immunoreactivity in the TH neurons might be interpreted as the light-touch sensitive DRG neurons being more vulnerable to certain stresses than other HAP1-ir sensory neurons. Future studies should include detailed nerve injury experiments to elucidate the effects of HAP1 on the vulnerability of DRG neurons.

It was recently reported that light-touch sensation is reduced in both extremities of SBMA patients (Grunseich and Fischbeck, 2015). SBMA patients show mostly intact other sensory and autonomic functions, whereas the motor functions are severely damaged (Sobue, 1995). Interestingly, motor neurons in spinal cord are also clearly devoid of HAP1- immunoreactivity (Islam et al., 2017). HAP1 can inhibit the nuclear translocation of gene products of a number of neurodegenerative diseases such as Huntington’s disease, Machado-Joseph disease and SBMA (Metzger et al., 2008; Takeshita et al., 2006, 2011). It has been hypothesized that HAP1 expression can raise the threshold of vulnerability to neurodegeneration and confer increased stability to neurons expressing HAP1, protecting against neurodegenerative apoptosis or cell death. Brain or spinal cord regions that are rich in HAP1, including limbic-hypothalamic regions of brain and the dorsal horn of the spinal cord, are usually spared from neurodegeneration. On the other hand, regions with little or no STB/HAP1 expression such as the neocortex, striatum, thalamus, cerebellum, and spinal motoneurons are major targets in the aforementioned neurodegenerative diseases (Fujinaga et al., 2004; Takeshita et al., 2006, 2011; Islam et al., 2012, 2017).

Although the mechanism by which the absence of HAP1 make the neurons vulnerable to neurodegeneration remains unknown, it has previously been hypothesized that HAP1 can sequester the pathological mutant molecules, trap the toxic aggregation in the cytoplasm and impede apoptosis-inducing nuclear translocation (Takeshita et al., 2006). It is possible that HAP1 could suppress the neurodegenerative process by protecting the subcellular cargo trafficking function, as it is known that HAP1 is associated with microtubule proteins (such as dynein or kinesin) and modulate the retrograde or anterograde transport of neuronal cargos between axonal/dendritic terminals and cell bodies (Li et al., 1998b; Goldstein and Yang, 2000; McGuire et al., 2006). In addition, subcellular augmentation of toxic forms of causative gene products has been reported to decrease the association of HAP1 with kinesin light chain and dynactin p150 (Smith et al., 2005; Gauthier et al., 2004). Although a consensus of further detailed immunohistochemical and physiological endorsements has yet to be obtained, our current results may suggest that the “HAP1 protection hypothesis,” which has previously been proposed for the brain and spinal cord in the central nervous system (Fujinaga et al., 2004; Takeshita et al., 2006, 2011; Islam et al., 2012, 2017), might also be applied to the DRG in the peripheral nervous system. HAP1 expression might increase the threshold of vulnerability to neurodegeneration and confer beneficial stability to different sensory neurons excluding the touch-sensitive mechanoreceptive ones in the DRG.

## 5 Conclusion

In conclusion, the current study is the first to elucidate the expression and distribution of HAP1 in the DRG throughout the cervical to sacral regions in adult male mice. The present study is also the first to clarify the characterization of HAP1- immunoreactivity in relation to the sensory neuron subpopulations. Our current results suggest the potential importance of HAP1 in pain transduction and proprioception. It will be of great interest to elucidate the pathophysiological roles of HAP1 in DRG.

Future experiments should include the evaluation of changes of HAP1 expression in DRG neuron subpopulations after specific injuries to the peripheral nerves. This will allow establishing the degree of participation of HAP1 in the mechanisms of nociception and mechanoreception.

## Author contributions

MNI and KS designed the experiments. MNI and MRJ performed Western blotting. MNI, NM, EM, AMMT, KN performed immunohistochemistry. MNI, TI, KM and AY performed the cell counting, tissue analyses and drawing Venn-diagram. MNI drafted the manuscript. KS critically revised the manuscript and supervised the study.

## Funding sources

This work was supported by Grants-in-Aid for Scientific Research of the Japan Society for the Promotion of Science (KAKENHI Grant Numbers 18K15006, 20K16108 to MNI and 19K02318 to AY) and Grants-in-Aid for Translational research of Yamaguchi University Hospital 2020 (to KN).

## Conflicts of interest

The authors have no conflicts of interest to declare.

## CRediT authorship contribution statement

**Md Nabiul Islam:** Conceptualization, Funding acquisition, Methodology, Investigation, Visualization, Formal analysis, Writing - original draft, Writing - review & editing. **Naoki Maeda:** Methodology, Investigation. **Emi Miyasato:** Methodology, Investigation. **Mir Rubayet Jahan:** Methodology, Formal analysis, Writing - review & editing. **Abu Md Mamun Tarif:** Methodology, Resources. **Taiga Ishino:** Validation, Software. **Kanako Nozaki:** Funding acquisition, Formal analysis. **Koh-hei Masumoto:** Data curation, Investigation. **Akie Yanai:** Funding acquisition, Validation, Investigation. **Koh Shinoda:** Funding acquisition, Supervision, Project administration, Visualization, Writing - review & editing.
